# 

**Published:** 1891-02-14

**Authors:** 


PRICE ONE PENNY.

229, Vol. IX.—February 14th, 1891.

[Registered at the General Post Office as a Newspaper for transmission in the United Kingdom and Abroad.]

WITH EXTRA NURSING SUPPLEMENT.

Per Annum, 6s. 6d.; post free.

Monthly Parts, 6d.; post free 8d.

Ditto per Annum, 8s., post free.

### THE HOSPITAL

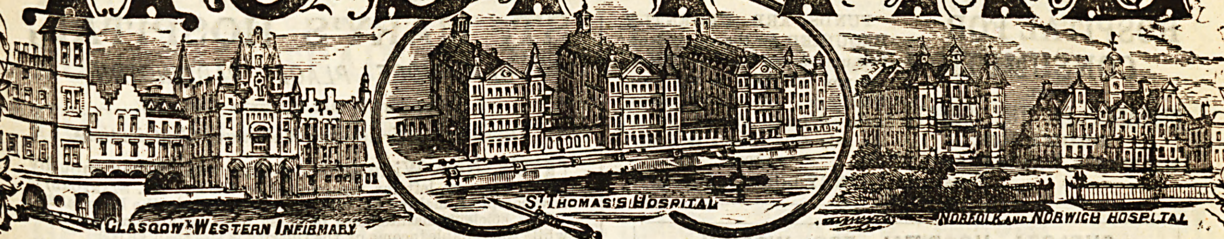

A WEEKLY JOURNAL OF

### Science, Medicine, Nursing and Philanthropy.

SATURDAY, FEBRUARY 14th, 1891.

**A Word for Poor Women** - 295

**Passing Topics.**—Legal Restrictions on Gifts to Charity - 296

**The Doctor's Armchair.**—A Counsel of Despair - 297

**The Microscope in Medicine.** By FRANK J. WETHERED, M.D.—V. Staining Sections - 298

**Recreation in Relation to the Health of the People.**—III. Working Up - 299

**Notes and News** - 300

**Everybody's Column** - 301

**Annotations.**—The Pressman, the Parson, and the Physiologist—The Sun and the Doctor—A House on Wheels - 302

**Scraps and Gleanings** - 301

**The Practitioner's Mirror and Retrospect:—**

MEDICINE.—Sciatica - 303

SURGERY.—Treatment of Malignant Diseases - 304

THERAPEUTICS.—Veratrum Viride - 305

NEW DRUGS, APPLIANCES, AND THINGS MEDICAL.—Dr. Koch—Sanitas Preparations - 305

**The Lords' Committee—General Charities** - 306

**The Student of Medicine.**—Editorial Notes—Notes from the Schools—Students' Medical Societies—Appointments—Vacancies—Athletics.

**EXTRA SUPPLEMENT—THE NURSING MIRROR.**

**En Passant.**—The Nurses' Co-operation—Keeping Christmas—Short Items—Sunderland Nursing Institute—Belfast Nurses—Birmingham District Nurses—Truro Nurses' Home
The Life of a Nurse - cvii
**Lectures on Surgical Ward Work and Nursing.** By ALEX. MILES, M.B. Edin., C.M., F.R.C.S.E. Lecture XIII.—Operations (*continued*) - cviii
**Appointments** - cviii
**The Midwives Bill** - cix
**For Reading to the Sick.**—Bad Nights - cix
**National Pension Fund for Nurses** - cix
**Notes from Australia** - cx
**A Winter's Holiday,** III.—The Third Week - cx
**Everybody's Opinion.**—The Death-rate in Typhoid—Infectious Hospitals - cxi
**Princess Christian's Daughter** - cxi
**Notes and Queries** - cxi
**Off Duty.**—Nurse Hilary (*continued*) - cxii
**Where to Go** - cxii
**Amusements and Relaxation** - cxii

